# Serum bile acid metabolic profiles in polycystic ovary syndrome: insights from targeted metabolomics and correlation with embryonic parameters

**DOI:** 10.3389/fmolb.2026.1730042

**Published:** 2026-05-04

**Authors:** Jialai Wang, Lirong Wang, Hang Ge, XinYu Hu, Zhenmin Si, Ying Yang, Fengjuan Li, Zhenyu Shi, Hui Chang

**Affiliations:** 1 The First Clinical Medical College, Heilongjiang University of Chinese Medicine, Harbin, China; 2 The First Affiliated Hospital of Zhejiang Chinese Medical University (Zhejiang Provincial Hospital of Traditional Chinese Medicine), Hangzhou, Zhejiang, China; 3 The First Clinical Medical College, Zhejiang Chinese Medical University, Hangzhou, Zhejiang, China; 4 Department of Obstetrics and Gynecology, First Affiliated Hospital of Heilongjiang University of Chinese Medicine, Harbin, China

**Keywords:** bile acids, embryonic parameters, metabolomics, polycystic ovary syndrome, ultra-high-performance liquid chromatography–tandem mass spectrometry

## Abstract

**Objective:**

Polycystic ovary syndrome (PCOS) is a complex endocrine disorder. In this study, we characterize serum bile acids (BAs) metabolic profiles in PCOS patients using targeted metabolomics and investigated their correlation with embryonic parameters, thereby elucidating the role of BAs in the pathogenesis of PCOS.

**Methods:**

We enrolled 20 PCOS patients undergoing *in vitro* fertilization-embryo transfer (IVF-ET) who met the Rotterdam criteria and 20 age-matched healthy controls. We recorded clinical baseline data and collected serum samples from all participants. The metabolic profiles of BAs were obtained by performing ultra-high-performance liquid chromatography–tandem mass spectrometry (UHPLC–MS/MS). Orthogonal partial least squares discriminant analysis (OPLS-DA) and receiver operating characteristic (ROC) curve analyses were performed to identify differential metabolites and evaluate their diagnostic value. Finally, we further analyzed the correlations between key differential metabolites and clinical indicators.

**Results:**

We identified 43 BA metabolites, including 22 upregulated and 21 downregulated species. We selected 11 key BA metabolites, of which six demonstrated diagnostic potential based on ROC curve analysis. We found negative correlations between these metabolites and embryonic parameters, although none of the correlations were statistically significant.

**Conclusion:**

Although targeted metabolomics is an exploratory tool, it is valuable for identifying potential diagnostic biomarkers in PCOS, offering preliminary novel insights into the pathophysiology of PCOS. The findings of this study suggest that targeted modulation of the metabolism of BA may represent an emerging and promising strategy for ameliorating metabolic and reproductive dysfunction in PCOS; however, these findings need to be validated in larger, independent cohorts.

## 1 Introduction

Polycystic ovary syndrome (PCOS) is a highly prevalent endocrine and metabolic disorder in women of reproductive age. PCOS is characterized by the dysregulation of gonadotropin secretion, hyperandrogenism, anovulation, and polycystic ovarian morphology, and its global prevalence is approximately 10% (Lizneva et al., 2016). PCOS is frequently accompanied by insulin resistance, obesity, and abnormalities in lipid metabolism (Anagnostis et al., 2018). These metabolic disturbances not only increase long-term risks associated with type-2 diabetes and cardiovascular diseases (Dapas and Dunaif, 2022) but also impair the reproductive health of affected women. Metabolic dysfunction may serve as an important link between reproductive abnormalities and long-term complications in PCOS patients.

The molecular mechanisms underlying metabolic dysfunction in PCOS have largely been elucidated. For example, studies on the amino acid metabolism have found significantly high plasma levels of branched-chain amino acids (BCAAs) in PCOS patients, with specific amino acids being correlated with greater risks of obesity, insulin resistance, and metabolic syndrome (Ye et al., 2022). Research on lipid metabolism has further identified BCAAs as prognostic factors for insulin resistance (Hajitarkhani et al., 2021).

Recent studies have found the novel role of bile acids (BAs) in modulating inflammatory responses, thereby contributing to the pathogenesis of PCOS (Ding et al., 2024). However, the precise mechanisms by which they play a role in PCOS remain poorly understood. BAs are terminal metabolites of cholesterol that not only facilitate the digestion and absorption of lipids but also regulate glucose and lipid metabolism, energy homeostasis, and immune-inflammatory responses via specific signaling pathways (Fuchs et al., 2025). Clinical studies have demonstrated significantly higher fasting serum BA levels in PCOS patients than in controls (Zhu et al., 2024). Additionally, high levels of circulating conjugated BAs have been positively correlated with hyperandrogenism in women with PCOS, manifesting in associated clinical symptoms (Zhang et al., 2019). By conducting a randomized controlled trial, researchers identified characteristic alterations in follicular fluid BA profiles in PCOS patients, and targeted metabolomics analysis revealed high levels of primary and conjugated BAs, suggesting that they may serve as biomarkers in the pathogenesis of PCOS (Yu et al., 2023). Moreover, high levels of BA transporter expression in ovarian tissue and the disrupted metabolism of BA may directly impair folliculogenesis and ovulation (Yang et al., 2021). Therefore, this study investigated BA metabolism to determine its pathological role in PCOS.

Metabolomics is a key component of systems biology and enables the comprehensive analysis of metabolic products to reflect overall metabolic status and functional changes. By integrating targeted metabolomics with bioinformatics, we elucidated the pathological mechanisms underlying BA metabolism in PCOS and its association with embryonic parameters, thereby providing novel therapeutic insights.

## 2 Materials and methods

### 2.1 Participants

In this study, the patient cohort comprised 20 individuals with PCOS undergoing *in vitro* fertilization-embryo transfer (IVF-ET), and the control group comprised 20 randomly selected age-matched healthy women. The diagnosis of PCOS adhered to the 2003 Rotterdam criteria (Rotterdam ESHRE/ASRM-Sponsored PCOS Consensus Workshop Group, 2004), requiring at least two of the following: (1) oligo-ovulation or anovulation; (2) presence of clinical (hirsutism, acne) or biochemical hyperandrogenism; (3) polycystic ovarian morphology, as determined by ultrasound examinations (≥12 follicles 2–9 mm in diameter in one or both ovaries and/or ovarian volume ≥10 mL). The exclusion criteria were as follows: (1) coexisting endocrine disorders (hyperprolactinemia, congenital adrenal hyperplasia, Cushing’s syndrome, and androgen-secreting tumors); (2) use of medications affecting outcomes within 3 months (oral contraceptives, hormonal agents, insulin sensitizers, and lipid-lowering drugs); (3) severe cardiopulmonary, hepatic, renal, or psychiatric conditions precluding participation. The study was approved by the Ethics Committee of the First Affiliated Hospital of Heilongjiang University of Chinese Medicine (approval no. KY2023-018), and all participants provided written informed consent.

### 2.2 Clinical data collection

Baseline clinical data were systematically recorded for all participants, including their height, weight, waist circumference, hip circumference, body mass index (BMI), and waist-to-hip ratio (WHR), along with their menstrual cycle characteristics. Fasting venous blood samples were collected on days 2–4 of the menstrual cycle or during withdrawal bleeding, processed into aliquots, and analyzed. Serum levels of luteinizing hormone (LH), follicle-stimulating hormone (FSH), LH/FSH ratio, estradiol (E2), testosterone (T), progesterone (P), fasting plasma glucose (FPG), fasting insulin (INS), triglycerides (TG), and low-density lipoprotein (LDL) were measured. Insulin resistance was quantified via homeostasis model assessment (HOMA-IR = [INS (mIU/L) × FPG (mmol/L)]/22.5). For participants undergoing IVF, several parameters were documented, including total oocytes, metaphase II (M2) oocytes, 2-pronuclei (2 PN) embryos, transferable embryos, and high-quality embryos.

### 2.3 Blood sample collection and preprocessing

For metabolomic analysis, another set of blood samples was left undisturbed at room temperature for 30 min and then centrifuged at 3,000 r/min for 10 min. The separated serum was aliquoted into EP tubes and stored at −80 °C in an ultra-low temperature freezer.

For metabolomic detection, 100 µL of plasma sample was first measured into an EP tube, and then 400 µL of ice-cold organic extraction solvent (methanol: acetonitrile, 1:1, v/v) was added. The mixture was immediately vortexed for 30 s and sonicated for 10 min in an ice-water bath. Next, the solution was incubated at −40 °C for 1 h to allow precipitation. Subsequently, the mixture was centrifuged at 12,000 r/min for 15 min at 4 °C. Finally, the supernatant was carefully transferred to LC-MS vials for subsequent UHPLC–MS/MS analysis.

### 2.4 UHPLC-MS/MS analysis

Bile acids were separated using a Vanquish ultra-high-performance liquid chromatography system coupled with a Waters ACQUITY UPLC BEH C18 column (150 × 2.1 mm, 1.7 μm, Waters Corp.). The mobile phase consisted of solvent A (5 mmol/L ammonium acetate in water) and solvent B (pure acetonitrile). The chromatographic conditions used for the analysis were that column temperature was maintained at 45 °C, autosampler temperature was set to 4 °C, and injection volume was 1 µL. Mass spectrometry was conducted on an Orbitrap Exploris 120 High-Resolution Mass Spectrometer in the parallel reaction monitoring (PRM) mode. The optimized ionization parameters included a spray voltage of +3,500 V (positive mode) and −3,200 V (negative mode), nitrogen gas flow rates of 40 (sheath gas) and 15 (auxiliary gas) arbitrary units, an auxiliary gas heater temperature at 350 °C, and a capillary temperature at 320 °C. The sweep gas was disabled (flow rate = 0) in this experiment.

### 2.5 Data processing and statistical analysis

The raw dataset comprising sample information, peak names, and peak intensities underwent log_10_ transformation before it was imported into the R ropls package. Supervised orthogonal partial least squares discriminant analysis (OPLS-DA) was applied to processed data (mean-centered and unit-variance-scaled) to identify metabolic differences between experimental and control groups. Variable importance in projection (VIP) scores were calculated to rank the contribution of each variable to the OPLS-DA model, with VIP >1.0 considered discriminatory. The model used seven-fold cross-validation as the default and underwent 200 response permutation tests to generate R^2^ and Q^2^ values for assessing model robustness and overfitting risk. R^2^Y = (0, 0.39) and Q^2^ = (0, −0.64). Generally, a Q^2^ value > 0.5 indicated good predictive capability for the model. Differential metabolites were determined based on the VIP score (VIP > 1.0) derived from the OPLS-DA model.

### 2.6 Statistical methods

Clinical data and laboratory test results were recorded in spreadsheets (Microsoft Excel) and analyzed using SPSS (version 27.0). Continuous variables following a normal distribution were presented as the mean ± standard deviation (SD), and between-group comparisons were performed using independent-samples t-tests. Data that did not follow a normal distribution were expressed as median (interquartile range), and group differences were assessed via the Mann–Whitney U test. The association between bile acid levels and embryo parameters was investigated using the Spearman rank correlation method. In multivariate analyses, including orthogonal partial least squares-discriminant analysis (OPLS-DA), known metabolic confounders that differed significantly between groups—BMI, homeostasis model assessment of insulin resistance (HOMA-IR), and TG—were incorporated as covariates. This adjustment was made to isolate the specific metabolic signature associated with PCOS independent of these common metabolic confounders. Statistical significance was set at *p* < 0.05.

### 2.7 Metabolomic data processing and quality control

To ensure data quality and decrease technical variation, we implemented a standardized preprocessing pipeline. We filtered raw peak areas and imputed any missing values using a random value between 0.1 and 0.5 times the minimum observed value for the corresponding metabolite. Subsequently, the data were log_10_-transformed and scaled to unit variance so that they followed a normal distribution and to give equal weight to all variables before multivariate statistical analysis.

An important step to avoid batch effects involved all samples being analyzed within a single, continuous analytical batch. Although a pooled quality control (QC) sample was not included in the final study cohort, instrument performance and stability were rigorously monitored by the analytical platform vendor throughout the data acquisition period using standard QC protocols.

Bile acids were quantified in the PRM mode using a predefined, targeted panel of metabolites. This targeted approach increased sensitivity and specificity for the metabolites of interest.

## 3 Results

### 3.1 Comparison of clinical characteristics

Significant differences were observed between the PCOS group and the normal control group in weight, BMI, waist circumference, and WHR (*p* < 0.05). The PCOS group had higher levels of LH, LH/FSH ratio, and T (*p* < 0.05), along with lower P (*p* < 0.05). In terms of glucose and lipid metabolism, FPG, INS, HOMA-IR, and TG were significantly higher in the PCOS group. Regarding embryo parameters, the PCOS group showed higher total oocyte retrieval and metaphase II (M2) oocytes (*p* < 0.05) (Table 1). These findings suggest that associations may be present, but the findings need validation in larger, independent cohorts.

**TABLE 1:** Clinical baseline characteristics of the participants.

Item	PCOS (n = 20)	Normal (n = 20)	*p-*value
Age (years)	32.50 ± 4.15	33.60 ± 3.55	0.373
Weight (kg)	65.03 ± 8.37	58.40 ± 9.44	0.024
Height (cm)	164.40 ± 3.89	164.35 ± 4.25	0.969
BMI (kg/m^2^)	24.00 ± 2.43	21.62 ± 3.37	0.015
Waist circumference (cm)	100.32 ± 11.36	90.23 ± 14.13	0.017
WHR	0.82 ± 0.01	0.80 ± 0.02	0.012
LH (IU/mL)	10.73 ± 3.29	4.64 ± 2.40	<0.001
FSH (IU/mL)	5.82 ± 1.53	6.32 ± 1.91	0.368
LH/FSH	1.88 ± 0.44	0.78 ± 0.43	<0.001
E2 (pg/mL)	47.3 ± 14.36	53.9 ± 20.73	0.244
T (ng/mL)	0.42 ± 0.17	0.29 ± 0.16	0.008
P (ng/mL)	0.21 ± 0.09	0.33 ± 0.17	−2.714
FPG (mmol/L)	5.48 ± 0.62	4.81 ± 0.43	<0.001
INS (µU/mL)	16.07 ± 7.16	10.87 ± 4.29	0.003
HOMA-IR	3.89 ± 1.65	2.31 ± 0.91	<0.001
TG (mmol/L)	2.08 ± 0.94	1.36 ± 0.78	<0.001
LDL (mmol/L)	3.28 ± 0.64	3.01 ± 0.44	0.126
Total oocytes(n)	16.00 (13.25–20.00)	13.00 (9.00–17.75)	0.033
2 PN(n)	12.00 (9.00–16.50)	10.00 (5.50–12.75)	0.063
M2 oocytes(n)	13.00 (11.25–17.75)	11.00 (7.25–15.00)	0.043
Transferable embryos(n)	5.50 (3.25–8.00)	7.00 (4.25–7.00)	0.237
High-quality embryos(n)	8.00 (6.00–13.25)	7.00 (5.00–10.00)	0.849

### 3.2 Data analysis and metabolite identification of BAs

**FIGURE 1:**
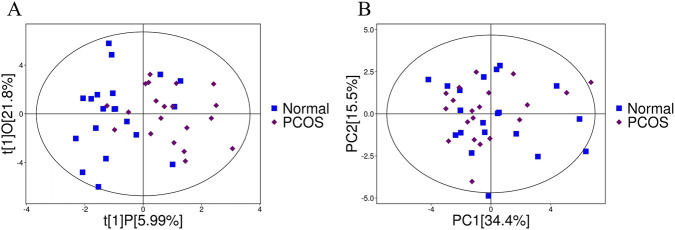
PCA and OPLS-DA score plots derived from BA metabolomic profiles comparing the PCOS and normal control groups. PCA **(A)** and OPLS-DA **(B)** were performed for the PCOS patients (purple circle) and the healthy controls (blue circle).

**FIGURE 2:**
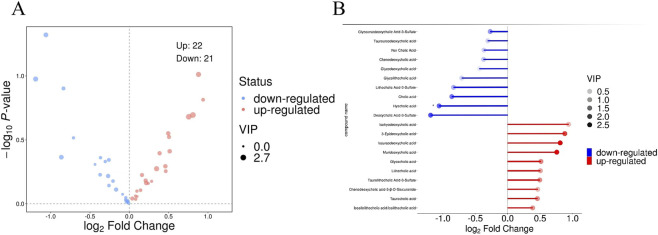
Differential expression analysis of BA metabolites. Volcano plot **(A)** and VIP–weighted bar plot **(B)**.

Principal component analysis (PCA) is an exploratory data analysis method, which was first used to construct an optimal projection plane that maps high-dimensional original data into a low-dimensional coordinate system with minimal loss of information, thereby concisely presenting the primary variance characteristics of the data (Figure 1A). Subsequently, OPLS-DA was applied for data modeling. Using this approach, we effectively separated orthogonal variations unrelated to classification variables while independently analyzing predictive variations, enabling a more accurate identification of differential metabolites associated with experimental groups and assessment of the strength of their correlations (Figure 1B). By conducting targeted metabolomics analysis, we identified 43 differential metabolites, including 21 downregulated and 22 upregulated species (Figure 2A). The top 10 upregulated and top 10 downregulated metabolites are illustrated in Figure 2B. As the key BA metabolites with VIP scores greater than 1, 11 compounds were obtained, which included isoursodeoxycholic acid (isoUDCA), murideoxycholic acid (MDCA), 3-epideoxycholic acid (βDCA), hyocholic acid (HCA), cholic acid (CA), glycoursodeoxycholic acid (GUDCA), glycocholic acid (GCA), deoxycholic acid-3-sulfate (DCA-3S), taurochenodeoxycholic acid (TCDCA), taurocholic acid (TCA), and taurolithocholic acid-3-sulfate (TLCA-3S) (Table 2).

**TABLE 2:** Quantitative detection results of BAs in the PCOS and control group.

Parameter	PCOS (n = 20)	Normal (n = 20)	VIP score	*p-*value
isoUDCA	32.52 (20.51–60.01)	62.98 (31.31–174.99)	2.70	0.202
MDCA	53.14 (29.94–88.67)	106.74 (43.54–234.61)	2.59	0.209
βDCA	85.11 (55.24–108.07)	87.53 (49.14–336.47)	1.94	0.097
HCA	8.28 (3.92–17.53)	4.28 (0.28–7.22)	1.46	0.048*
CA	44.22 (20.81–123.32)	20.89 (17.33–65.19)	1.34	0.432
GUDCA	28.17 (7.82–51.11)	21.91 (12.96–39.29)	1.90	0.532
GCA	57.98 (33.70–259.77)	124.48 (50.84–254.32)	1.14	0.388
DCA-3S	4.34 (0.27–14.58)	5.96 (0.23–8.08)	1.73	0.106
TCDCA	37.18 (19.68–148.92)	66.04 (27.31–138.28)	1.09	0.689
TCA	6.42 (2.26–33.05)	14.22 (7.29–50.13)	1.05	0.509
TLCA-3S	23.25 (0.28–50.91)	38.43 (20.44–64.37)	1.06	0.281

* indicates that the P-value is less than 0.05, which represents a statistically significant difference.

### 3.3 ROC curve analysis and expression levels of key BAs

**FIGURE 3:**
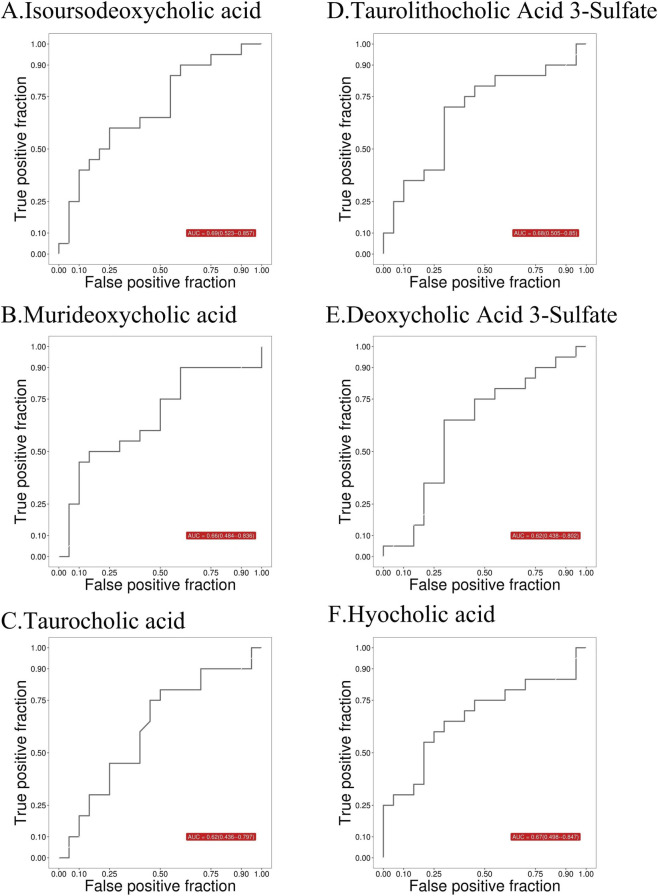
ROC curves of the six key metabolites in the PCOS group. **(A)** Isoursodeoxycholic acid. **(B)** Murideoxycholic acid. **(C)** Taurocholic acid. **(D)** Taurolithocholic acid 3-sulfate. **(E)** Deoxycholic acid 3-sulfate. **(F)** Hyocholic acid.

**FIGURE 4:**
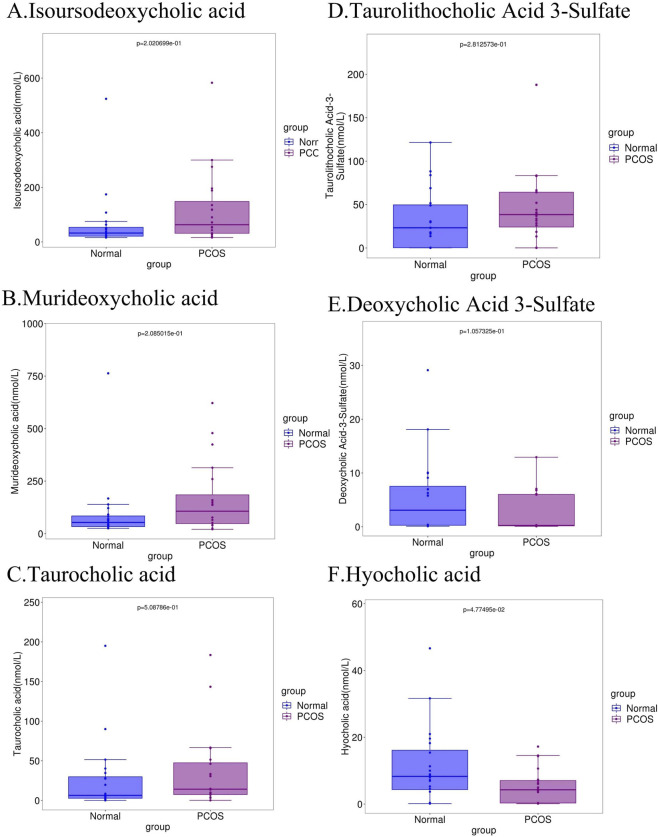
Box plots showing differences in BA levels between the PCOS and control groups. **(A)** Isoursodeoxycholic acid. **(B)** Murideoxycholic acid. **(C)** Taurocholic acid. **(D)** Taurolithocholic acid 3-Sulfate. **(E)** Deoxycholic acid 3-Sulfate. **(F)** Hyocholic acid.

The predictive sensitivity of the screened key metabolites for distinguishing PCOS from control groups was evaluated using the area under the receiver operating characteristic curve (AUC), with an AUC > 0.6 considered to suggest the presence of diagnostic value. Among the upregulated BAs, isoUDCA (AUC = 0.69; 95% CI: 0.523–0.875), MDCA (AUC = 0.66; 95% CI: 0.484–0.836), TCA (AUC = 0.62; 95% CI: 0.438–0.802), and TLCA-3S (AUC = 0.60; 95% CI: 0.414–0.776) demonstrated significant discriminatory ability. For downregulated BAs, DCA-3S (AUC = 0.68; 95% CI: 0.505–0.85) and HCA (AUC = 0.67; 95% CI: 0.498–0.847) met the threshold (Figure 3). Subsequent analysis of the expression levels of these six key BAs revealed that, compared to the control group, the PCOS group exhibited a significantly lower serum concentration of HCA (*p* < 0.05) (Figure 4). These findings indicate that BA metabolism may play a role in PCOS, but further investigation is needed to confirm this speculation.

### 3.4 Correlation between key BA metabolites and clinical parameters

Based on the ROC curve analysis, six key BA metabolites were identified. To investigate their associations with embryonic parameters, Spearman’s correlation analysis was conducted. The results indicated that most of these BAs exhibited negative correlations with embryonic parameters, although none of the correlations were statistically significant (Table 3).

**TABLE 3:** Association between BA levels and embryo parameters in IVF cycles.

BA	Embryo parameter	Pearson’s r	*p-*value
isoUDCA	Total oocytes	−0.201	0.397
	2 PN	−0.297	0.203
	M2 oocytes	−0.310	0.184
	Transferable embryos	−0.297	0.204
	High-quality embryos	−0.278	0.235
MDCA	Total oocytes	−0.142	0.550
	2 PN	−0.249	0.289
	M2 oocytes	−0.257	0.273
	Transferable embryos	−0.255	0.278
	High-quality embryos	−0.230	0.329
TCA	Total oocytes	−0.164	0.490
	2 PN	−0.079	0.740
	M2 oocytes	−0.130	0.586
	Transferable embryos	0.290	0.215
	High-quality embryos	0.072	0.762
TLCA-3S	Total oocytes	−0.039	0.869
	2 PN	−0.159	0.502
	M2 oocytes	−0.023	0.924
	Transferable embryos	−0.053	0.824
	High-quality embryos	−0.201	0.395
DCA-3S	Total oocytes	−0.279	0.233
	2 PN	−0.242	0.304
	M2 oocytes	−0.193	0.415
	Transferable embryos	−0.155	0.515
	High-quality embryos	−0.328	0.158
HCA	Total oocytes	−0.072	0.764
	2 PN	−0.198	0.403
	M2 oocytes	−0.244	0.301
	Transferable embryos	−0.425	0.062
	High-quality embryos	−0.230	0.329

## 4 Discussion

The etiology of PCOS is extremely complex. We investigated serum BA metabolic profiles in a cohort of PCOS patients undergoing IVF-ET and compared these profiles to those of healthy controls using targeted metabolomics. As the sample size was small (n = 40), the observed differences and associations described below are preliminary and exploratory; therefore, larger-scale studies are needed to validate our findings. Consequently, no mechanistic conclusions can be drawn from this study. Our analysis suggests potential alterations in the BA profile of PCOS patients relative to the control group. PCOS patients exhibited alterations in their BA profile, with significantly lower serum levels of isoUDCA, MDCA, TCA, TLCA-3S, GUDCA, GCA, and TCDCA, while levels of DCA-3S, CA, and HCA were significantly higher. Further ROC analysis demonstrated diagnostic value for isoUDCA, MDCA, TCA, TLCA-3S, DCA-3S, and HCA in distinguishing PCOS patients from healthy individuals, indicating their value as candidate biomarkers.

BAs function as signaling molecules that activate multiple nuclear and membrane receptor-mediated pathways in various tissues, thereby regulating glucose and lipid homeostasis, inflammatory responses, and energy expenditure (Fiorucci et al., 2021). The levels of BAs in PCOS patients, including those of cholic acid and ursodeoxycholic acid, are negatively correlated with androgen concentrations, which may aggravate the clinical manifestations of hyperandrogenism (Qi et al., 2019). Altered BA metabolism in PCOS, particularly aberrant synthesis and metabolic pathways in the liver and intestine, may be closely associated with the development of insulin resistance (Staels and Fonseca, 2009). In our study, although the results were not statistically significant, we observed that isoUDCA, an epimer of ursodeoxycholic acid (UDCA) with hydrophilic and cytoprotective properties (Purucker et al., 2001), was present at lower levels in PCOS patients than in healthy controls, suggesting that it may be involved in the pathogenesis of PCOS; however, further investigation is needed to confirm this speculation. Its downregulation may imply a weakened cytoprotective effect on liver, intestinal, or ovarian tissues, rendering these more susceptible to damage from metabolic disturbances or inflammatory stress; this could be a potential factor involved in the pathogenesis of PCOS.

As shown in animal studies, the intraduodenal administration of TCA markedly inhibits dietary lipid absorption and attenuates postprandial triglyceride fluctuations, thereby improving lipid metabolism (Farr et al., 2020). Xu et al. (2022) consistently reported that TCA alters the composition of gut microbiota and BA profiles while activating TGR5 and FXR signaling pathways to enhance the transport and reabsorption of BAs. The lower (though not significant) TCA levels observed in our PCOS patients align with this regulatory axis. The reduction in TCA may impair the regulation of lipid metabolism and maintenance of intestinal microecological homeostasis mediated via the TGR5/FXR pathways, which could partially explain the common abnormalities in lipid metabolism and propensity for insulin resistance in PCOS patients. This association requires further assessment in larger-scale studies.

Glycocholic acid (GCA) is a potent suppressor of hyperactive immune responses. Its mechanism involves the activation of the FXR receptor, which inhibits pro-inflammatory cytokine secretion in lipopolysaccharide-stimulated murine macrophages. This demonstrates that GCA can serve as a naturally derived anti-inflammatory agent capable of modulating immune function (Ge et al., 2023). Yamamura et al. (2025) revealed that fecal GCA levels exhibit a significant positive correlation with BMI, with high GCA groups showing substantially greater obesity risk. Genes encoding BA deconjugation enzymes were downregulated in these high-GCA cohorts, suggesting that GCA may influence the development of obesity by altering the composition of the gut microbiota. GCA supplementation can also alleviate hepatic cholestasis, hepatic steatosis, and intestinal damage induced by high-pectin diets. This effect is characterized by an increase in hepatic total BA concentrations and significantly upregulated expression of the FXR gene. The GCA-mediated activation of FXR promotes the expression of BA synthesis genes such as CYP7A1 and CYP27A1 and enhances the activity of BA transporters such as BSEP and NTCP, thereby restoring BA homeostasis (Yao et al., 2024). The non-significant trend toward lower GCA levels in our PCOS patients suggests a potential disturbance in this anti-inflammatory or metabolic regulatory pathway.

Hyocholic acid (HCA), a primary bile acid, exhibits unique receptor effects by simultaneously activating both TGR5 and FXR. This dual agonism increases the production and secretion of glucagon-like peptide-1 (GLP-1) in intestinal endocrine cells, thereby improving insulin sensitivity (Sun et al., 2021). A cohort study demonstrated that HCA levels can predict the future risk of diabetes as diabetic patients have significantly lower concentrations of fecal HCA (Wang et al., 2023). The glucose-lowering mechanism of HCA involves the activation of TGR5 and inhibition of FXR, thus increasing the secretion of GLP-1 from enteroendocrine cells. Additionally, the HCA/TGR5 signaling pathway in the ileum regulates postprandial GLP-1 expression through interactions between the gut microbiota and host metabolism (Anhê et al., 2019). Our data showed significantly higher serum HCA levels in PCOS patients (P = 0.048). This observed increase in HCA in PCOS may represent a compensatory adjustment or dysregulation that disrupts FXR-mediated suppression of hepatic gluconeogenesis and TGR5-dependent stimulation of GLP-1 secretion, thereby exacerbating insulin resistance (IR). The level of DCA-3S showed a non-significant reduction in PCOS patients in our study, which differs from some previous observations and requires further validation. Following the activation of FXR, NF-κB/Nrf2 signaling is inhibited, which attenuates the release of pro-inflammatory cytokines (Wang et al., 2024). FXR further regulates BA transporters such as Bsep, Ntcp, and Ugt1a1, along with metabolic enzymes such as Cyp7a1, thus alleviating inflammation and oxidative stress (Liu et al., 2022). Similarly, HCA suppresses NF-κB phosphorylation, nuclear translocation, and transcriptional activity, downregulating inflammatory genes (IL-1β, IL-6, and TNF-α) and inhibiting the LPS-induced activation of AKT, which exert anti-inflammatory effects (Kuang et al., 2023).

In summary, this preliminary investigation describes potential perturbations in the BA profile of PCOS patients. The changes observed in specific BAs, such as isoUDCA, TCA, GCA, and HCA, offer exploratory hypotheses regarding their involvement in PCOS-related metabolic and inflammatory dysregulation. However, the absence of statistically significant correlations indicates that the results should be interpreted with caution. Future studies with larger sample sizes are needed to validate these alterations in the BA profile, determine their clinical relevance, and identify any causal role in the pathophysiology of PCOS.

## 5 Limitations

Although this study preliminarily revealed the potential role of BA metabolism in diseases through targeted metabolomics analysis, it had certain limitations. First, only the change in the level of HCA was statistically significant. Although another five types of BAs (UDCA, MDCA, TCA, TLCA-3S, and DCA-3S) showed diagnostic value through the ROC curve, the differences in their expressions were not significant. This may be related to insufficient statistical power due to a small sample size, and the statistical power may also be limited by the sensitivity of serum metabolite detection or biological variation. Although these five types of BA did not show significant differences, they have potential associations with key receptors (such as FXR and TGR5) in metabolic pathways, and they may participate in the disease process by regulating glycolipid metabolism or inflammatory signals in a coordinate way. For example, the hydrophilic cytoprotective effect of UDCA and the regulatory effect of taurocholic acid on the intestinal flora suggest that these metabolites may serve as markers or regulatory targets for disease progression.

Second, as this was an observational and cross-sectional study, causality cannot be inferred. The observed associations between altered BA profiles and PCOS phenotype could not establish whether these changes were a cause or a consequence of the metabolic disturbances of the syndrome. Third, the lack of measurement of the key BA synthesis biomarker 7α-hydroxy-4-cholesten-3-one (C4) limits the ability to delineate whether the observed serum BA profile stems primarily from dysregulated hepatic synthesis or altered enterohepatic circulation. Finally, a single serum metabolomic snapshot cannot accurately capture potential diurnal or physiological fluctuations in BA levels, and the targeted approach may have missed other relevant metabolites.

In this study, the statistical analysis had several limitations. Multiple comparison correction was applied using the Benjamini–Hochberg procedure to control the false discovery rate (FDR) for the 43 BA metabolites measured. However, after adjustment, most of the resulting Q-values did not reach the conventional threshold for statistical significance (FDR <0.05), with the reported adjusted Q-value being 0.99. Therefore, the interpretation of differential metabolites relied primarily on VIP scores from OPLS-DA and unadjusted nominal p-values.

This outcome may be attributed to insufficient statistical power, given that the sample size was relatively small (n = 20 per group). Strict correction for multiple testing further reduced the ability to detect metabolites with modest but biologically meaningful effect sizes while limiting Type I errors. Therefore, all candidate metabolites identified in this study should be considered preliminary and exploratory biomarkers rather than robust diagnostic indicators.

Future investigations with larger independent cohorts are required to validate these findings and allow reliable statistical inference based on appropriately corrected significance thresholds.

In future research, the sample size needs to be increased, and multi-omics technologies, such as intestinal flora sequencing and fecal bile acid profile analysis, need to be incorporated along with functional experiments to validate the clinical significance of these BAs. This integrated approach is essential to comprehensively elucidate the pathological mechanisms underlying the BA metabolic network in the disease. Longitudinal studies incorporating C4 measurement are also needed to validate these findings, determine directionality, and reveal the intricate mechanistic links within the gut–liver axis in PCOS.

## 6 Conclusion

This study analyzed the metabolic characteristics of BAs in the serum of 20 PCOS patients and 20 healthy controls through targeted metabolomics. We identified 43 BAs, 11 of which were key metabolites and 6 had diagnostic value. PCOS patients showed abnormalities in metabolic indices such as weight and BMI, as well as sex hormones such as LH and T, with significantly reduced serum levels of hyocholic acid. Although the key BAs were mostly negatively correlated with embryo parameters, the differences were not statistically significant. Our findings indicated that targeted metabolomics can be used to identify potential biomarkers for PCOS; this can provide new insights into the pathological mechanisms underlying PCOS and the strategy of targeting BA metabolism to improve reproductive function.

## Data Availability

The original contributions presented in the study are publicly available. This data can be found here: https://www.ebi.ac.uk/metabolights/MTBLS14387 accession number MTBLS14387.

## References

[B1] Anagnostis P. Tarlatzis B. C. Kauffman R. P. (2018). Polycystic ovarian syndrome (PCOS): long-term metabolic consequences. Metabolism Clinical Experimental 86, 33–43. 10.1016/j.metabol.2017.09.016 29024702

[B2] Anhê F. F. Nachbar R. T. Varin T. V. Trottier J. Dudonné S. Le Barz M. (2019). Treatment with camu camu (Myrciaria dubia) prevents obesity by altering the gut microbiota and increasing energy expenditure in diet-induced obese mice. Gut 68 (3), 453–464. 10.1136/gutjnl-2017-315565 30064988

[B3] Dapas M. Dunaif A. (2022). Deconstructing a syndrome: genomic insights into PCOS causal mechanisms and classification. Endocr. Reviews 43 (6), 927–965. 10.1210/endrev/bnace001 35026001 PMC9695127

[B4] Ding S. Li W. Xiong X. Si M. Yun C. Wang Y. (2024). Bile acids in follicular fluid: potential new therapeutic targets and predictive markers for women with diminished ovarian reserve. J. Ovarian Research 17 (1), 250. 10.1186/s13048-024-01573-3 39702491 PMC11657518

[B5] Farr S. Stankovic B. Hoffman S. Masoudpoor H. Baker C. Taher J. (2020). Bile acid treatment and FXR agonism lower postprandial lipemia in mice. Am. Journal Physiology. Gastrointest. Liver Physiology 318 (4), G682–G693. 10.1152/ajpgi.00386.2018 32003602

[B6] Fiorucci S. Distrutti E. Carino A. Zampella A. Biagioli M. (2021). Bile acids and their receptors in metabolic disorders. Prog. Lipid Research 82, 101094. 10.1016/j.plipres.2021.101094 33636214

[B7] Fuchs C. D. Simbrunner B. Baumgartner M. Campbell C. Reiberger T. Trauner M. (2025). Bile acid metabolism and signalling in liver disease. J. Hepatology 82 (1), 134–153. 10.1016/j.jhep.2024.09.032 39349254

[B8] Ge X. Huang S. Ren C. Zhao L. (2023). Taurocholic acid and glycocholic acid inhibit inflammation and activate farnesoid X receptor expression in LPS-stimulated zebrafish and macrophages. Switzerl. 28 (5), 2005. 10.3390/molecules28052005 36903252 PMC10003765

[B9] Hajitarkhani S. Moini A. Hafezi M. Shahhoseini M. Alizadeh A. (2021). Differences in gene expression of enzymes involved in branched-chain amino acid metabolism of abdominal subcutaneous adipose tissue between pregnant women with and without PCOS. Taiwan. Journal Obstetrics & Gynecology 60 (2), 290–294. 10.1016/j.tjog.2020.12.008 33678329

[B10] Kuang J. Wang J. Li Y. Li M. Zhao M. Ge K. (2023). Hyodeoxycholic acid alleviates non-alcoholic fatty liver disease through modulating the gut-liver axis. Cell Metab. 35 (10), 1752–1766.e8. 10.1016/j.cmet.2023.07.011 37591244

[B11] Liu J. Liu J. Meng C. Huang C. Liu F. Xia C. (2022). Oleanolic acid alleviates ANIT-Induced cholestatic liver injury by activating fxr and Nrf2 pathways to ameliorate disordered bile acids homeostasis. Phytomedicine International Journal Phytotherapy Phytopharmacology 102, 154173. 10.1016/j.phymed.2022.154173 35605478

[B12] Lizneva D. Suturina L. Walker W. Brakta S. Gavrilova-Jordan L. Azziz R. (2016). Criteria, prevalence, and phenotypes of polycystic ovary syndrome. Fertil. Sterility 106 (1), 6–15. 10.1016/j.fertnstert.2016.05.003 27233760

[B13] Purucker E. Marschall H. U. Winograd R. Matern S. (2001). Metabolism and effects on cholestasis of isoursodeoxycholic and ursodeoxycholic acids in bile duct ligated rats. Biochimica biophysica acta 1526 (1), 44–52. 10.1016/s0304-4165(01)00096-4 11287121

[B14] Qi X. Yun C. Sun L. Xia J. Wu Q. Wang Y. (2019). Gut microbiota-bile acid-interleukin-22 axis orchestrates polycystic ovary syndrome. Nat. Medicine 25 (8), 1225–1233. 10.1038/s41591-019-0509-0 31332392 PMC7376369

[B15] Rotterdam ESHRE/ASRM-Sponsored PCOS consensus workshop group (2004). Revised 2003 consensus on diagnostic criteria and long-term health risks related to polycystic ovary syndrome (PCOS). Hum. Reproduction Oxf. Engl. 19 (1), 41–47. 10.1093/humrep/deh098 14688154

[B16] Staels B. Fonseca V. A. (2009). Bile acids and metabolic regulation: mechanisms and clinical responses to bile acid sequestration. Diabetes Care 32 (Suppl. 2), S237–S245. 10.2337/dc09-S355 19875558 PMC2811459

[B17] Sun L. Cai J. Gonzalez F. J. (2021). The role of farnesoid X receptor in metabolic diseases, and gastrointestinal and liver cancer. Nat. Reviews. Gastroenterology & Hepatology 18 (5), 335–347. 10.1038/s41575-020-00404-2 33568795

[B18] Wang Q. Lin H. Shen C. Zhang M. Wang X. Yuan M. (2023). Gut microbiota regulates postprandial GLP-1 response *via* ileal bile acid-TGR5 signaling. Gut Microbes 15 (2), 2274124. 10.1080/19490976.2023.2274124 37942583 PMC10730136

[B19] Wang M. Q. Zhang K. H. Liu F. L. Zhou R. Zeng Y. Chen A. L. (2024). Wedelolactone alleviates cholestatic liver injury by regulating FXR-bile acid-NF-κB/NRF2 axis to reduce bile acid accumulation and its subsequent inflammation and oxidative stress. Phytomedicine International Journal Phytotherapy Phytopharmacology 122, 155124. 10.1016/j.phymed.2023.155124 38014837

[B20] Xu J. Xie S. Chi S. Zhang S. Cao J. Tan B. (2022). Protective effects of taurocholic acid on excessive hepatic lipid accumulation *via* regulation of bile acid metabolism in grouper. Food & Function 13 (5), 3050–3062. 10.1039/d1fo04085e 35199809

[B21] Yamamura R. Okubo R. Ukawa S. Nakamura K. Okada E. Nakagawa T. (2025). Increased fecal glycocholic acid levels correlate with obesity in conjunction with the depletion of archaea: the dosanco health study. J. Nutritional Biochemistry 139, 109846. 10.1016/j.jnutbio.2025.109846 39863085

[B22] Yang X. Wu R. Qi D. Fu L. Song T. Wang Y. (2021). Profile of bile acid metabolomics in the follicular fluid of PCOS patients. Metabolites 11 (12), 845. 10.3390/metabo1120845 34940603 PMC8703527

[B23] Yao S. Ren S. Cai C. Cao X. Shi Y. Wu P. (2024). Glycocholic acid supplementation improved growth performance and alleviated tissue damage in the liver and intestine in *Pelteobagrus fulvidraco* fed a high-pectin diet. Fish Physiology Biochemistry 50 (1), 41–57. 10.1007/s10695-022-01148-3 36454392

[B24] Ye Z. Zhang C. Wang S. Zhang Y. Li R. Zhao Y. (2022). Amino acid signatures in relation to polycystic ovary syndrome and increased risk of different metabolic disturbances. Reprod. Biomedicine Online 44 (4), 737–746. 10.1016/j.rbmo.2021.11.012 35131170

[B25] Yu J. Zhang Y. Zhu Y. Li Y. Lin S. Liu W. (2023). Circulating bile acid profile characteristics in PCOS patients and the role of bile acids in predicting the pathogenesis of PCOS. Front. Endocrinology 14, 1239276. 10.3389/fendo.2023.1239276 37693357 PMC10484098

[B26] Zhang B. Shen S. Gu T. Hong T. Liu J. Sun J. (2019). Increased circulating conjugated primary bile acids are associated with hyperandrogenism in women with polycystic ovary syndrome. J. Steroid Biochemistry Molecular Biology 189, 171–175. 10.1016/j.jsbmb.2019.03.005 30849463

[B27] Zhu Y. Lin S. Zhang Y. Yu J. Fu J. Li Y. (2024). Altered bile acids profile is a risk factor for hyperandrogenism in lean women with PCOS: a case control study. Sci. Rep. 14 (1), 26215. 10.1038/s41598-024-77645-7 39482365 PMC11528117

